# Searching for nests of the invasive Asian hornet (*Vespa velutina*) using radio-telemetry

**DOI:** 10.1038/s42003-018-0092-9

**Published:** 2018-07-04

**Authors:** Peter J. Kennedy, Scott M. Ford, Juliette Poidatz, Denis Thiéry, Juliet L. Osborne

**Affiliations:** 1 Environment and Sustainability Institute, University of Exeter, Penryn Campus, Penryn, Cornwall TR10 9FE UK; 2 INRA, UMR 1065 Santé et Agroécologie du Vignoble, 71 avenue Edouard Bourlaux, 33882 Villenave d’Ornon Cedex, France

## Abstract

Asian hornets (*Vespa velutina*) are voracious predators of bees, and are the latest emerging threat to managed and wild pollinator populations in Europe. To prevent establishment or reduce the rate of spread of *V. velutina*, early detection and destruction of nests is considered the only option. Detection is difficult as their nests are well hidden and flying hornets are difficult to follow over long distances. We address this challenge by tracking individual *V. velutina* workers flying back to their nests using radio telemetry for the first time, finding five previously undiscovered nests, up to 1.33 km from hornet release points. Hornets can fly with 0.28 g tags if the tag:hornet ratio is less than 0.8. This method offers a step-change in options to tackle the spread of this invader, providing an efficient means of finding *V. velutina* nests in complex environments to manage this emerging threat to pollinators.

## Abstract (web summary)

Peter Kennedy et al. report on the successful tracking of Asian hornets, an invasive species in Europe that threatens bee populations. Their method enabled them to track individual hornets over long distances and follow them to locate five previously undiscovered nests in France and the Channel Islands.

## Introduction

The Asian hornet or yellow-legged hornet (*Vespa velutina nigrithorax* Lepeletier) is an invasive alien species^1, 2^ that poses an immediate and substantial threat to European pollinators^3, 4^. Originating from Asia, it was first recorded in Europe in 2005^5^, introduced via trade through south-western France in or before 2004^6^. It has spread across France, into northern Spain, Portugal, Italy, Germany, Mallorca Island, the Channel Islands^7, 8^ and was recorded for the first time on the UK mainland in September 2016^9^. The invasion front on the European continent is estimated to have spread at approx. 60–78 km per year^10, 11^, with occasional nests reported 200 km ahead of the front suggesting accidental human-mediated transport of founding queens^4^. This is likely to have consequences not only for beekeeping and honey production, but also for crop and wild flower pollination. The hornets live in large eusocial colonies producing up to 15,000 individuals over the colony’s lifetime and several hundred gynes in autumn^10^. They are voracious predators of insects, showing a preference for Hymenoptera including honeybees, wild bees and wasps^12, 13^. The foragers hawk at the entrance of honeybee hives, snatching and killing returning honeybee foragers^14^. The bee colony responds by shutting down foraging effort so the colony is not only weakened by levels of predation but may starve from lack of food^15, 16^. To date there has been no scientific evaluation of the overall impact of *V. velutina* on honeybee colony survival but, in France, beekeepers estimate that they have lost between 5 and 80% of honeybee colonies (average 30%) where *V. velutina* has established^3^. This is similar to the scale of losses resulting from the spread of *Varroa destructor* mites across Europe in the 1990s^17^. Of additional concern is that *V. velutina* preys on wild insects, and the level of damage to pollinator populations and pollination services could be extensive but is so far unquantified^4^. The speed of establishment^11, 18^ indicates that the only means of containing or curtailing the spread is to locate and destroy colonies at the invasion front as early in the season as possible, before reproductives (gynes and males) are produced^3, 19^.

There is thus an urgent need to develop a method to locate *V. velutina* nests quickly and efficiently so that they can be destroyed if required. Most nest recovery currently depends on visual searches with limited efficiency^18^ since *V. velutina* nests are well hidden, often positioned high in trees or in dense scrub, and often in urban areas^10, 20^. The most effective visual search method involves recording the ‘vanishing direction’ in which hornets fly away from foraging sites, and then homing in on the *V. velutina* nest by setting bait traps in a series of steps closing in on the source—a method that can take days and several people (R. Hogge, Jersey, personal communication). Harmonic radar, used successfully to track individual flying bees over hundreds of metres^21, 22^, is unsuitable for *V. velutina* because it relies on tracking in open, flat landscapes, as vegetation obscures the signal. Milanesio et al.^23, 24^ have developed another harmonic radar system, bespoke for *V. velutina*, with a wider beam width. It is able to identify preferred flight directions of returning hornets but signal strength and range are constrained in wooded and urban environments, and the equipment’s mobility is restricted by its size. No successful nest searches have been reported to date. Thermal cameras mounted on drones are currently being tested^25^, but may only be useful in the vicinity of the nest (<100 m). We considered using the hand-held RECCO™ radar system that has been used on insects such as beetles and dragonflies^26–30^. The insect tags are light (0.03 g, since they are passive so do not require a battery), but their range is less than 100 m, and can be as short as 12 m (depending on tag orientation) (P. Kennedy, unpublished data), limiting its likely use given that *V. velutina* can fly over a kilometre from their nests on foraging trips^9^. Radio-telemetry has been successfully used to track vertebrates for over 50 years^31, 32^ but examples of its use for tracking flying insects, reviewed by Kissling et al.^29^, are more limited owing to how the weight (largely due to the battery) and antennal length of even the smallest tags may still impede flight. The smallest tags from Biotrack Ltd, UK, weigh 0.22 g (Pip19/Ag190 tag) and 0.28 g (PicoPip/Ag337 tag), with antenna length of 10 cm, and a battery life of 4–12 days. Despite their weight these tags do have advantages over radar because they are individually traceable, even in dense and complex environments (not relying on line-of-sight), and detectable to a range of ~800 m for PicoPip/Ag337 tags, and ~375 m for Pip19/Ag190 tags. They are commercially available giving the opportunity to develop a technique useable by a range of stakeholders and non-specialists to find *V. velutina* nests to manage the risk of this invasive species. The technique of using tagged individuals to find conspecifics, in species that are known to aggregate into groups, has been termed the Judas technique^33^. This technique has been employed against mammals^34^, birds^35^ and fish^36^, but only recently against an insect pest (the Coconut rhinoceros beetle, *Oryctes thinonceros*)^37^ and here for the first time to our knowledge against a social insect.

Since *V. velutina* workers are robust fliers weighing approximately 0.140–0.475 g and are adapted to carry large prey items^10^ we chose to test these radio tags to track flying hornets. For nest detection we only require hornets to fly back to their nests and we do not assume that hornets are flying without energetic penalties^29^. So, here we demonstrate that *V. velutina* workers can be individually tracked using radio telemetry to find their nests in complex environments, using a new method of attaching some of the smallest individually identifiable radio tags available^29^. This represents a major step forward in developing contingency plans to manage this emerging threat to pollinators. It is also a breakthrough for investigating the hornet’s ecology in greater detail, opening up the possibility of developing further pest management options.

## Results

### *Vespa velutina* flight capability when carrying radio tags

**Fig. 1:**
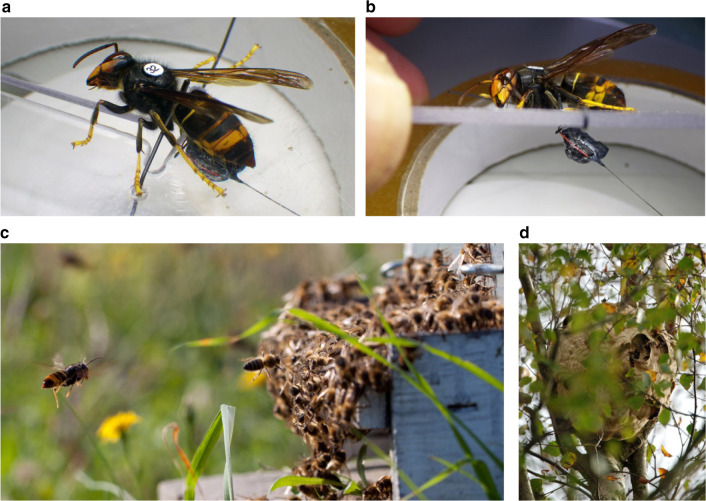
Tracking *V. velutina* from foraging site to nest. *Vespa velutina* workers were caught when hawking outside of hives, foraging around plants for nectar or honeydew, or at bait stations, were then fitted with VHF radio-telemetry tags, released near their point of capture and tracked to their nests. **a** Worker hornet (ID = W32; weight = 0.48 g) restrained to a Perspex plate while attaching PicoPip Ag337 tag (ID = N347; configuration Option A; weight = 0.30 g). **b** Side view of hornet (ID = W32) with PicoPip Ag337 tag. **c** Asian hornet hawking outside of a honeybee hive in France. **d** *Vespa velutina* nest in a Silver birch (*Betula pendula*) tree in a garden near Trinity, Jersey. All photographs by P. Kennedy, except (**c**) by Karine Monceau

*Vespa velutina* workers were caught foraging at two field sites (South West France and Jersey) and those selected for testing ranged in weight from 0.229 to 0.490 g (Supplementary Table 1). We designed a new method for attaching a radio tag (Pip19/Ag190 or PicoPip/Ag337), or tag mimic (dummy; see Methods) to a *V. velutina* worker. Unlike most other insect tracking studies where tags are attached dorsally^29^ (although not for dragonflies^38, 39^), the most successful method for attaching a radio tag to *V. velutina* was to attach it ventrally (without using glue, tied with a cotton thread loop across the petiole) (Fig. 1a, b, Supplementary Fig 1). In this configuration the hornet could both walk and fly following tag attachment. No hornets flew well with dorsal tag attachment. Importantly, the hornets were not launched immediately into flight but were given 10–20 min to adjust to the presence of the tag.

After 10–20 min adjustment, we tested whether each hornet could fly with the tag in place, both inside an insect-proof outdoor flight cage and freely outside the cage to confirm whether observation of flight performance within the cage was an indicator of successful free flight. Of the 36 tagged hornets assessed both in the cage and field, 17 flew well in the cage and all of these flew well in the field (Table 1). Poor flyers in the cage were usually also poor flyers in the field (12 out of 19), although seven hornets flew poorly in the cage but went on to fly well outside. There was thus a highly significant association between flight performance in the cage and outside (Fishers exact test: two-tailed *P* = 0.0000452). So successful flight in the cage, after tag attachment, can be used as an indicator that the tagged hornet is likely to fly well when released, thus reducing the chances of failed tracking attempts, and loss of tags.

**Table 1:** Flight performances of tagged hornets in the field cage versus free-flying in the field

		In a field cage		
		Good flyers	Poor flyers	Total
In the field	Good flyers	17	7	24
	Poor flyers	0	12	12
	Total	17	19	36

Flight performance of hornets fitted with radio-telemetry tags, or hand-made mimics of such tags, were judged first in a flight cage 3 m x 3 m x 2 m and subsequently on release in an open field. Hornets demonstrating repeated horizontal flights or ascending flights after initial release from an elevated position (1.3 m inside the field cage; 1.6 m in the field) were categorised as good flyers. Hornets predominately demonstrating descending flights were categorised as poor flyers. Analysed via two-by-two contingency table using Fisher’s exact test; two-tailed *p* value = 0.0000452 showing strong association between flight performance in both arenas

**Fig. 2:**
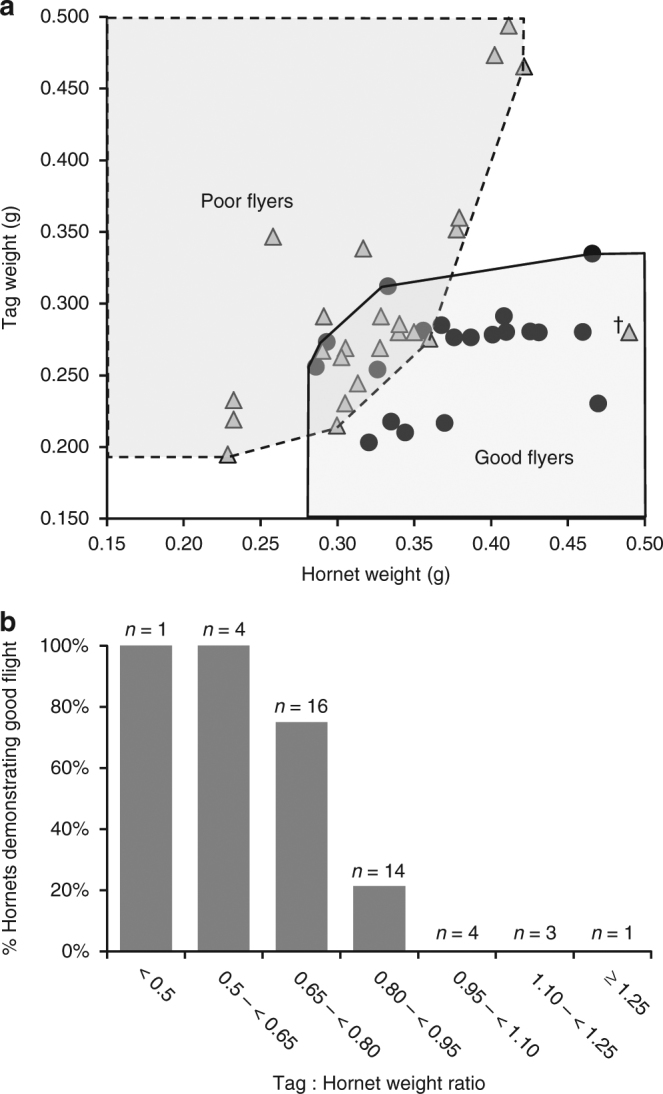
Flight performance of tagged hornets. **a** Hornet and tag weight combinations resulting in hornets, on release into a field cage (3 m x 3 m x 2 m), being categorised as good (closed black dot) (*n* = 24) or poor (triangle) (*n* = 20) flyers based on flight behaviour. A hornet (cross symbol), with fresh weight = 0.49 g and tag weight = 0.28 g, is considered an outlier as caught & held in a hornet trap for over 5 h before its flight performance was assessed. **b** Percent of hornets (out of 43 assessed), grouped according to their tag:hornet weight ratio, demonstrating good flight performance on release into a field cage (3 m x 3 m x 2 m) or flight room (2 m x 2 m 2 m)

For each *V. velutina* worker, we recorded how tag:hornet weight ratio correlated with flight capability. The range of tags tested weighed 0.195–0.494 g. The heaviest tag with which a hornet flew well was 0.334 g (dummy tag, Fig. 2a). Most hornets (81%) carrying tags less than 80% of their body weight flew well, but above this threshold good flight was only observed for 14% of hornets (Fig. 2b). These results show that ensuring a tag:hornet ratio less than 0.8 is likely to result in successful tracking. Since large workers (>0.35 g) could fly with the PicoPip/Ag337 tag (0.28 g), and because of this tag’s longer detection range (up to 800 m) than the Pip19/Ag190, we focussed on using the larger tag to test whether we could find *V. velutina* nests.

### Radio-tracking foraging *V. velutina* back to their nests

Eight of the *V. velutina* workers that were tagged with PicoPip/Ag377 tags (Fig. 2a, b, Supplementary Table 2), and whose flight capability was checked in the cage (tag:hornet weight <0.8), were released near their point of capture and their flight activity tracked using a Sika radio tracking receiver (Biotrack Ltd, UK) scanning for the signal from the active tag. The tagged hornet was thus followed on its flight path back to the nest. When lack of variation in signal for 5–10 min suggested an individual had stopped, a visual inspection was made to search for a potential nest, usually within the leaf canopy of a tree, or for evidence of a foraging site.

**Fig. 3:**
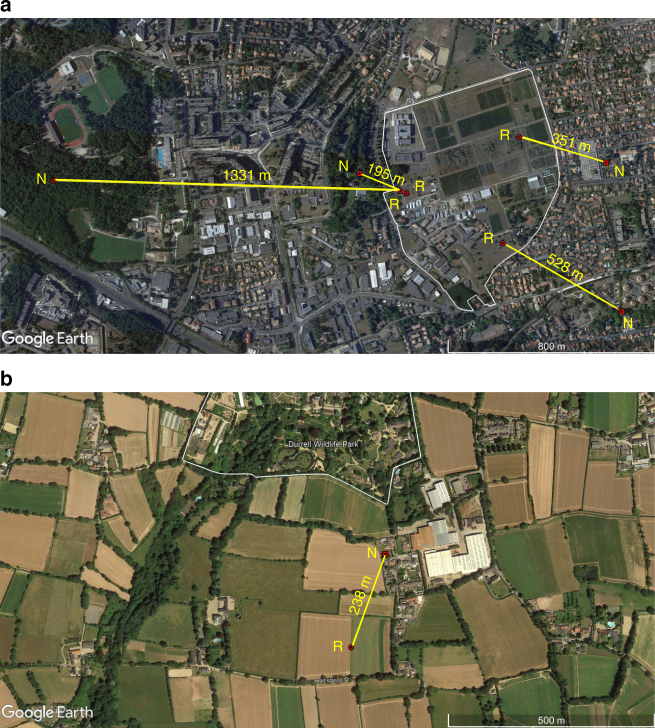
Tracked hornets’ nest locations. Captured hornet workers were fitted with a PicoPip Ag337 radio tag, released (R) in the vicinity of where they had been caught, and tracked via radio-telemetry on their return flights to their nests (N). Yellow lines and distances indicated relate to the shortest distance between a release point of a tracked hornet and her corresponding nest, rather than flight paths taken. **a** From the grounds of INRA Bordeaux-Aquitaine (white polygon) in Villenave d’Ornon, a suburb of Bordeaux. **b** Near Trinity and Durrell Wildlife Park (Les Augrès Manor; white polygon) on Jersey. GPS coordinates of the hornet release and nest locations were transferred onto satellite maps from GoogleEarthPro. Data on radio-tracked locations and waypoints are given in Supplementary Tables 2 and 3

Six *V. velutina* workers were tagged and released in France and two in Jersey, and all were successfully tracked to between 45 m and 1331 m from their release point. Five of these hornets were tracked to their previously undiscovered nests (4 in France and 1 in Jersey: Fig. 3)—a 100% success rate of tracking and a 63% success rate of nest detection. The nests found ranged from 195 to 1331 m (average = 529 m) from the tagged hornets’ release point (where they had been originally foraging). For the three hornets that were successfully tracked, but where no nest was detected, this was *not* due to signal detection failure (Supplementary Tables 2, 3): two hornets stopped in inaccessible places, and the third hornet ceased to fly after it was caught in extensive heavy rain and sought shelter overnight in a nearby tree.

Two hornets were tagged with the lighter Pip19/Ag190 tags but flew away rapidly following release, and their radio signal was subsequently lost soon after they were no longer visually discernible. This tag was therefore not suitable when used by a single tracking team, due to its limited range and the flight speed of the tagged hornets.

In terms of effort required to find a nest, the average time spent tracking a hornet from its release to nest detection was 92 ± 37 min (mean ± standard deviation) for two people, although it would be feasible for one person to complete the task. Tagged hornets actually flew at 2–4 m s^−1^, but search time includes hornets stopping, and time taken for the tracking team to home in on the signals. In France, four nests were found over 3 days. Here the land-cover was predominately urban. It was thus not feasible to follow the radio signals directly by compass direction, but required the tracking team to follow roads and pathways, triangulating on the signal. Nevertheless, the radio signal was strong enough in this highly complex environment to find the nests in trees at large distances from the hornet foraging site. The nests were all found high in trees (above 5 m). In Jersey, the land-cover was predominately farmland, but the hornet nest was found in a tree in a private garden (Fig. 1d). The tagged hornets did not necessarily go straight back to the nest after being released: five of the hornets were tracked to foraging sites, feeding on ivy, willow or mulberry, before returning to their nests. One hornet visited a sweet chestnut tree (*Castanea sativa*), 625 m from its nest, on the day after it had been tracked to its nest—demonstrating that the tag attachment had not seriously compromised the insect’s ability to fly.

One of the main advantages of this new radio-tracking technique is speed and efficiency: In Jersey, in September and October 2017, a team of 3–4 beekeepers/officials spent ~5 days finding each *V. velutina* nest (they found 10) using vanishing directions and visual searches. Our radio-tracking could take 1–2 people under 2–3 h, from hornet capture under optimum conditions, to find each nest. Tracking thus only needs 25% of the person resources, and importantly would be achieved in a fraction of the time, giving the added advantage of earlier nest destruction before reproductives emerge, reducing chances of *V. velutina* spread the following year.

## Discussion

Our study clearly demonstrates that radio-telemetry can be used to successfully find *V. velutina* nests in complex, heterogeneous environments. All the hornets tagged and released with 0.28 g PicoPip/Ag377 tags were tracked successfully, resulting in the discovery of five nests in urban (France) and farmed (Jersey) landscapes. These nests were found within hours of locating and capturing the foraging hornets; so this technique would make an efficient and reliable tool to complement a contingency plan focussed on detection and destruction of *V. velutina* nests at the invasion front^40^. Having a method to detect *V. velutina* nests is not only important for invasion management, but also enables their ecology and behaviour to be examined in greater detail, at different stages of the invasion timeline, so that the impact of this species on wild and managed insects can be measured.

We have shown the opportunities and constraints of using this technique. *Vespa velutina* workers weighing 0.356–0.475 g could effectively fly with a 0.28 mg PicoPip/Ag377 tag, tied to the petiole and positioned ventrally. Hornets could fly with tags weighing up to 80% of their body weight. For the technique to be successful it is best to choose the largest hornet workers possible, avoiding those >0.500 g fresh weight (potentially gynes which are unlikely to return to the nest)^10^, and give the hornets time to recover from the attachment procedure in an environment in which they can adapt their flight behaviour for at least 20 min before they are released. The average weight of hornet workers increases through the season as the colony develops, although there is considerable variation between colonies and individuals depending on resources, environment and latitude^10^. If the hornet workers are smaller (0.28–0.35 g), for example earlier in the season, then 0.22 mg Pip19/Ag190 tags could be used. As these lighter tags have a shorter detection range (<375 m), multiple tracking teams (multiple receivers) may be required, but this is so far untested. The use of the radio-telemetry may be limited by the availability of large hornet workers in areas where the *V. velutina* population is only recently established since the density of nests, and consequently workers, will be low. On the other hand, only one suitable hornet is required to find a nest.

Tackling *V. velutina* by early and efficient nest detection is the best opportunity to halt or slow invasion in European countries (such as the UK) where it is not yet well established. Governments and beekeepers across the EU are looking for methods to slow the spread of this pest species and scientists have documented the urgent need for research on early nest detection^19, 41^. Nest density in Andernos-les-Bains, France, rose from zero to 12.3 km^−2^ within 10 years^42^ (although this included embryo nests which subsequently may not have reached maturity) and, with no control, Keeling et al.^18^ predict that there will be over 50,000 nests across England and Wales within 10 years, reaching carrying capacity in 20 years. So, while these predictions could be interpreted as a worst case scenario, they indicate that speed of action is critical. We are at the beginning of the invasive process in the UK and northern Europe so the opportunity to curtail the invasion and limit impact is short^9, 11^. The costs of control and management rise exponentially as an invasive species spreads^17^ so there are clear benefits to early intervention, which will result in long-term cost saving. While a full valuation requires further data, we have estimated what our new nest detection protocol would save in lost profits to beekeepers in the UK, using a similar approach to that used for *Varroa destructor* mites^17^. Based on there being ~250,000 managed UK honeybee colonies, worth ~£600 per annum each to the UK economy in honey production and crop pollination^17^, *V. velutina* establishment could result in *at least* another 20–30% losses (on top of normal 10–15% annual losses) costing the UK economy ~£30–45million. If the nest detection protocol doubled the number of nests detected, *V. velutina* nest density and honeybee colony losses could be reduced by 30% ^11^, saving at least £10–£15 million in lost profits to the UK each year. This does not include savings in beekeepers’ time to manage *V. velutina* attacks, the cost of beekeepers giving up keeping bees in response to *V. velutina*, or the costs saved to the authorities managing this pest as it spreads. The radio-telemetry equipment itself would need to be purchased, at a current 2018 cost for reciever and antenna of less than £2000 plus approximately £140 per radio tag (subject to any additional tax, currency conversion and delivery costs). The more immediate benefits of finding nests reliably using radio-tracking is that it will result in needing 10% of the person power, and nests will be found in a fraction of the time taken for traditional visual searches, giving the added advantage of earlier *V. velutina* nest destruction before reproductives disperse and establish nests the following year. The figures are cautiously calculated, and do not include the value of damage to wild pollinator populations, or the indirect effects on crop pollination that may ensue.

The impact of *V. velutina* on wild pollinators is so far unquantified, and losses are less recoverable than for honeybees because wild species are not managed, so there is little chance of mitigation. For this reason the potential ecological impact of *V. velutina*, beyond the economic, should not be underestimated, and this new method of tracking hornets to their nests should be rapidly incorporated in *V. velutina* invasion management plans across Europe.

## Methods

### Study overview

The study was carried out on workers of *Vespa velutina nigrithorax* (also known as yellow-legged or Asian hornets), found foraging in SW France and in Jersey. At the time of the study (July and September 2017) *V. velutina* nests were building towards maturity with hundreds to thousands of workers searching for insect prey to feed developing brood in their colonies. The purpose of the technique developed was to find nests before reproductives (gynes and males) were produced (typically between September and December^10^).

### Field sites

Methods were developed and tested within the grounds of INRA Bordeaux-Aquitaine, in the southern suburbs of Bordeaux (Villenave d’Ornon, Gironde, southwestern France). Method development (cold anaesthesia, tag attachment and recovery post-handling) took place from 17 to 24 July 2017; tests of tagged hornet flight performance took place from 25 to 27 July and 13 to 21 September 2017. Tagged hornets were tracked in Villenave d’Ornon on 27 July and between 13 and 21 September 2017 under warm, dry conditions (average daily temperatures = min 10.4 °C, max 22.2 °C; average daily rainfall = 1.9 mm; average wind speed = 3.5 m s^−1^). We also tested the technique on *V. velutina* in Jersey (Crown Dependency of the UK, located 14 miles from the north west coast of France), which has a much lower density of hornets as they have newly established here^8^. Testing and tracking was carried out between 25 and 28 September 2017 at the States of Jersey Department of Environment (Howard Davis Farm, La Route de la Trinité, Trinity, Jersey JE3 5JP) and near to points of capture in and around the Durrell Wildlife Park (Trinity) and New Zealand Avenue, St Saviour. These parishes are predominately rural with some semi-urban development (average daily temperature = min 13.8 °C, max 18.8 °C; average daily rainfall = 2.8 mm; average wind speed = 4.2 m s^−1^).

### Radio tags

The Pip19 (with Ag190 battery) and PicoPip (with Ag337 battery) radio tags (both from Biotrack Ltd, UK) were deemed the most suitable as they were among the lightest, smallest VHF tags available. They are sold in various configurations differing in location of the battery relative to the tag electronic circuit board and orientation of their aerial. Initial attempts utilised tags in Biotrack’s Option A configuration (with batteries on top of circuit boards generating a shorter, fatter tag: 8 mm long × 5 mm wide × 4 mm tall) but subsequently tags in Biotrack’s Option C configuration (with battery fixed at the end the circuit board generating a longer, slimmer tag: 13 mm long × 5 mm wide × 2 mm tall) were preferred as these fitted better under the abdomen of *a V. velutina* worker. All tags had a 100 mm aerial in the same plane as the length of the tag (hence trailing behind it), although these could be shortened but with a loss in range in doing so. Pip19/Ag190 tags weighed 0.222 g (SD ± 0.016 g; range = 0.195–0.245 g; includes a small 7 mg metal loop glued to the tag for attachment as described below) and were activated by cutting a connecting wire on the side of the tag. Once activated, Pip19 tags with a pulse length of 15 ms and pulse frequency of 28–32 ppm (pulses per minute) had a manufacturer’s expected lifespan of 4 days. PicoPip/Ag337 tags weighed 0.280 g (SD ± 0.012 g; range = 0.256–0.312 g; including the small 7 mg metal attachment loop) and were activated by removal of a magnet linked to an integral reed switch. Once activated, PicoPip tags with a pulse length of 19–20 ms and a pulse rate of 39–46 ppm had a manufacturer’s expected lifespan of 12 days. Reed switches come with a slight weight penalty but were preferred during testing as such tags could be readily activated and deactivated when reusing tags on different hornets (a factor less relevant to tracking as hornets were infrequently re-caught following field release). Reed switches were not utilised with Pip19 tags as the weight penalty was considered proportionally more substantial. Testing of representative Pip19 and PicoPip tags in a flat open landscape (Predannack airfield, Ruan Major, UK) indicated expected detection ranges of approx. 250 m (max. 375 m) and approx. 500 m (max. 817 m), respectively.

Each tag operated at a specific frequency within the band designated for wildlife telemetry within that country (in France = 150.xxx MHz; in UK including Jersey = 173.xxx MHz). Tags of different frequencies were used to ensure activated tags could be distinguished in the field. The frequencies were preprogrammed into a Sika radio tracking receiver (138–174 MHz band width; Biotrack Ltd, UK), fine-tuned to each individual tag, and signal detection confirmed with a suitable Yagi antenna (Biotrack Ltd, UK) before release of a tagged individual.

To test the capabilities of hornets to carry tags of differing weights, we utilised Pip19 and PicoPip tags (either active or with expired batteries) and further expanded the weight range tested by utilising home-made mimics of such tags, made from short lengths of electric mains core wrapped in insulating tape and with a thin 100 mm aerial, to an equivalent size, shape and relevant weight.

All tags had a very small metal wire loop (resistant to bite action by the hornet) glued on to front edge of the tag, allowing the tag and aerial to trail behind from this attachment point (see below for attachment to hornet). As live tags were encased by the manufacturer in either varnish or Plastidip, the tags were made not only water resistant but avoided the attached wire loop short-circuiting the tag electronics. Each tag was marked with either a unique identifier provided by the manufacturer or by the addition of unique mark (e.g. numbered and coloured honeybee queen marking disc glued to dummy tags). Before use, each tag was weighed, with their metal wire attachment loop in place, on an electronic laboratory balance.

### Attachment of the radio tag

*Vespa velutina* hornets were caught in an insect net while hawking outside beehives, foraging around a willow tree, or foraging at artificial bait stations (prawn-baited in Bordeaux; Trappit^®^ wasp attractant baited in Jersey). Once caught, individual hornets were transferred via 50 ml Falcon centrifuge tubes (with ventilation hole/slit in cap) to the laboratory where they were weighed on an electronic laboratory balance. To avoid testing or tracking *V. velutina* gynes, queens or males, female individuals weighing <0.500 g were selected^10^. Selected individuals were cold anaesthetised by embedding the Falcon tube, with hornet, to its full length in crushed ice for a minimum of 10 min or until the hornet no longer showed discernible movement (max. 12 min). Once anaesthetised, a hornet would be secured to a bespoke restraining plate (Supplementary Fig. 1). The abdomen of an anaesthetised hornet would be carefully manoeuvred under the wire tie on the plate, ensuring wings and legs were free of entrapment, before the wire ends were pulled tight, lowering the hoop, between abdomen and thorax, across the hornet’s petiole. The 20 mm distance between drilled holes and the stiffness of the wire ensured that the wire would restrain the hornet to the plate without damaging it, even when the wire was pulled very tight. The wire ends were secured by bending them over opposite edges of the restraining plate. Thin cotton sowing thread was next fed though the head of the T-shaped cut in the restraining plate, over the hornet’s petiole, and back down through the head of the T-shaped cut in the restraining plate (avoiding legs, wings and wire tie). The thread was drawn sufficiently tight and thread ends tied in a knot to produce a thread loop around the petiole that was loose enough to allow free movement but close enough to prevent a hornet from reaching and biting the thread loop. The remaining ends of cotton thread were then passed though the small metal wire loop previously attached to the radio tags and tied in a further tight knot. Knots were further secured by a small dap of superglue, and surplus lengths of thread removed close to the final knot. An anaesthetised *V. velutina* worker would partially recover within the short time (<5 min) needed to attach a tag but would remain secure on the restraining plate. To ensure subsequent identification of individuals, a unique coloured and numbered honeybee queen marking disc was glued (Loctite Super Glue Power Flex Gel Control) on to the dorsal surface of the thorax of each hornet.

The above tag arrangement and attachment permitted a radio tag to be secured to a central ventral point of the hornet, with the attachment point beyond reach of the hornet’s mandibles, but nevertheless allowing sufficient movement in the tag enabling the hornet to manoeuvre past obstacles when on a surface. In flight, the tag and aerial would trail below and slightly behind the hornet with little direct impact on flight (earlier attempts at fitting tags to the dorsal side of the thorax proved top-heavy and led to hornets flipping over and falling to ground; unpublished data).

Once marked and tagged, a hornet was released into a ventilated recovery cage (0.6 m x 0.6 m x 0.7 m) by loosening the wire tie and guiding the hornet and tag along the T-shaped slit until free from the restraining plate and secured in the cage. Hornets were given 10 min to recover from anaesthesia and handling within the cage in which food (honey, fruit syrup and water) was provided ad libitum. This recovery period also permitted released hornets to begin to adapt flight behaviour to take account of the weight and size of attached tags. Ad hoc observations during this period also permitted checks to confirm tags were attached in a manner permitting the hornets to walk and fly within the confines of the cage.

### Testing whether tagged *V. velutina* can fly

Flight performance of 47 tagged hornets was assessed in an outdoor flight cage (3 m x 3 m x 2 m insect-proof and pollination netted cage from Diatex SAS, France; ref. PE16/13.28), or on field release near point of initial capture (37 hornets in both, 7 in the flight cage only, and 3 on field release only; see Supplementary Table 1). For ease of release, the recovery cage was placed inside the outdoor flight cage on a raised platform. On completion of the recovery period, a selected hornet was placed on the roof of the recovery cage and released to assess its flight performance over the subsequent 10 min. A released hornet would either walk or fly to the edge of the roof and launch into the air towards the perimeter of the flight cage. From the approx. 1.3 m vantage of the recovery cage roof, a hornet would descend to the ground, achieve near horizontal flight, demonstrate slight ascending flights, or variants between these. As well as an opportunity to assess flight performance, this period in the flight cage was also a further opportunity for hornets to adapt to the presence of radio tags. Hornets that landed on the ground were either guided towards the flight cage netting to allow them to climb to a height, or lifted back on to the recovery cage roof. Flight performance was assessed based on repeated flights or flight attempts within the 10 min period, and rated on a scale of 1−5 (1 = sharp descending flights only or no flights; 2 = mainly descending flights; 3 = both horizontal and descending flights; 4 = mainly horizontal flights; 5 = ascending and strong horizontal flights). On a conservative basis, hornets rated 4 or 5 were subsequently categorised as good flyers and hornets rated 1 to 3 were categorised as poor flyers within the flight cage.

On completion of the flight performance assessment in the flight cage, hornets were transferred to an open field location. Using blunt forceps, a hornet would be placed on a gloved hand held approx. 1.6 m above the ground. The hornet was allowed to fly off when ready or, if remained stationary for over 5 min, was gently encouraged to depart by slight movement of the forceps near the hornet. A hornet was permitted three attempts to fly beyond reach. If all three attempts were descending towards the ground, the hornet was classified as a poor flyer and recovered. If the hornet flew beyond reach (e.g. into a tree at >12 m from the release site, or beyond a distance where it was visually discernible), it was categorised a good flyer. Hornets that were fitted with active radio tags and subsequently tracked were included in these flight assessments.

### Tracking tagged *V. velutina* to their nests

Ten tagged *V. velutina* workers prepared for tracking were transferred to a field location, close to where they had been caught: seven hornets in France and three hornets in Jersey. Two hornets were fitted with Pip19 tags and eight hornets with PicoPip tags. The functioning of the active tags was verified by tuning the Sika radio tracking receiver to the preprogrammed channel and checking for a detectable signal via Sika receiver with Yagi antenna prior to release.

*A V. velutina* worker selected for release was placed on an elevated surface (either the roof of a parked car or on a gloved hand; both approx. 1.6 m above the ground). As previously during flight performance assessments, each hornet was given no more than three attempts to fly from an elevated location. All ten *V. velutina* workers flew either to a nearby tree or flew beyond a distance where they were visually discernible. In all cases, the release time, GPS coordinates (latitude and longitude) of the release site and vanishing direction (i.e. compass bearing) from the release point were recorded. The visually determined vanishing direction was also confirmed as the direction providing the strongest signal from the tag attached to the released hornet.

The tracking team, made up of a Sika receiver operator and data recorder, would quickly relocate along the vanishing direction, checking for the direction of strongest signal reception. The tracking team was restricted to using public roads or paths, municipal recreational areas, or land where prior permission to enter had been sought. Consequently, the tracking team could not follow the hornets’ flight paths directly but had to triangulate from accessible locations. Variation in the detected signal’s strength or direction from a location would indicate whether a tagged hornet was likely stationary or in flight. Although principally a direction indicator, adjustment of the gain/sensitivity of the Sika receiver would indicate whether a hornet was close, on occasions confirmed by visual sightings of a tagged hornet. The time, signal direction and GPS coordinates of various waypoints along a tracking route were recorded as well as any observational notes such as foraging activity around nectar-rich food sources (e.g. flowering ivy covered trees). When the detected signal was deduced to converge on a single location from various waypoints over a period of 5–10 min, the location in question was visually checked through binoculars looking for evidence of a nest (usually within the leaf canopy of a tree), concentrated hornet traffic (suggesting a concealed nest) or the tagged hornet, or else to determine whether it was a likely foraging site (evidenced by abundant nectar-rich flowers such as ivy; and associated hornet activity). Such searching continued until either observed or daylight faded. When a nest was found, its location was recorded and the local hosts (INRA in France; and Department of Environment in Jersey) informed to enable them to activate the locally appropriate management plan and limit the impact of *V. velutina*.

### Analysis

Each captured hornet was weighed and all behavioural information recorded after tagging, through to the location of nests. For a range of tag weights and hornet weights, the tag:hornet weight ratio was calculated to determine the threshold relating to good flight performance (Fig. 2). Distances between release point and nest location were calculated from the latitude and longitude of the two points (Fig. 3). All data are included in the Supplementary Tables.

### Statistical analysis

Flight performance was divided into two classes: poor and good flyers, for tests both within the cage and outside. A Fisher’s exact test on the two-by-two contingency table (Table 1) was used to test for association between performances of individuals inside and outside the cage, to explore whether flight within the cage was a good indicator of flight outside the cage.

### Data availability

The authors declare that all data supporting the findings of this study are available within the article and Supplementary Tables 1–3.

## Electronic supplementary material

Supplementary Information
