# Supplementary material for: Searching for nests of the invasive Asian hornet (Vespa velutina) using radio-telemetry
Source: Commun Biol. 2018 Jul 4;1:88. doi: 10.1038/s42003-018-0092-9 (PMC6123801; doi:10.1038/s42003-018-0092-9)
Supplement: Supplementary file 1 — Supplementary Information [file 42003_2018_92_MOESM1_ESM.pdf]

Supplementary Figure 1. Restraining plate for attaching a radio tag to a hornet

(a) **(b)**

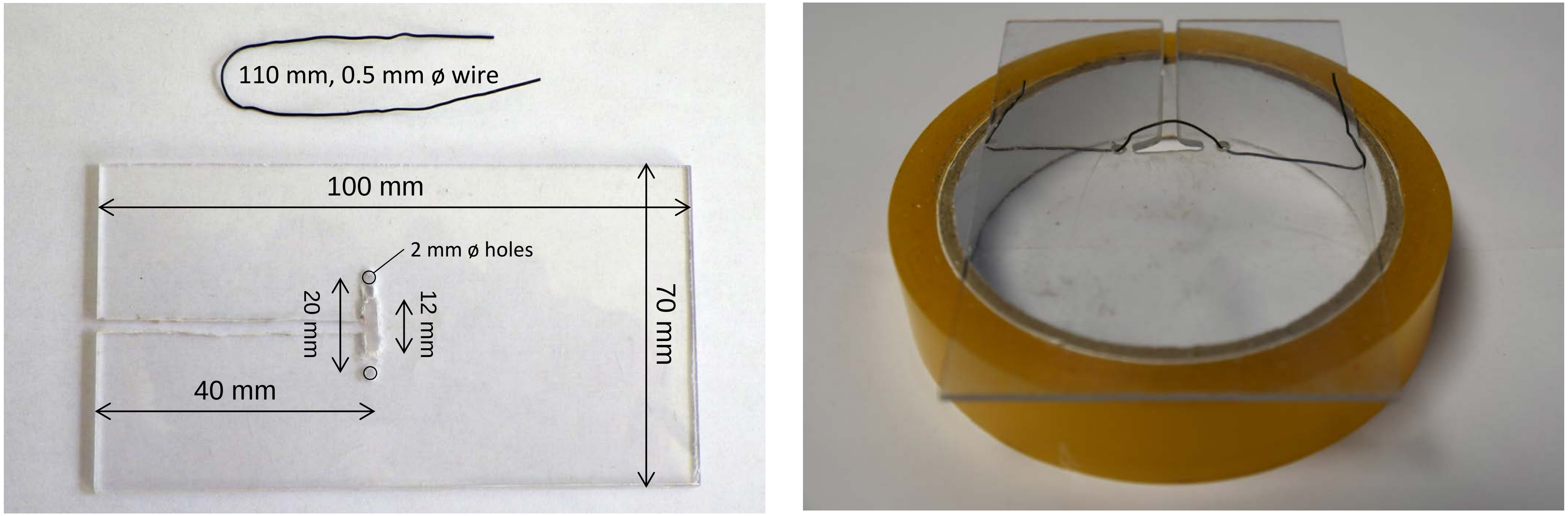

Restraining plate: **(a)** top view with dimensions, and **(b)** with wire tie in place. The plate is made from a 100 mm x 70 mm rectangle of 1.7 mm thick acrylic sheet, with a 2 mm slit extending centrally from a short edge into a 40 mm long and 12 mm wide T-shape. Two 2 mm diameter holes were drilled 20 mm apart, either side of the 12 mm wide 'head' of the cut T. A wire tie (110 mm long; 0.5 mm diameter) was bent into a u-shaped hoop and its ends passed through the two drilled holes in the restraining plate. Figure annotated in Microsoft PowerPoint, using photographs taken by P.Kennedy.

Supplementary Table 1. Tagged worker hornet's flight performance.

Hornet		Tag			Tag:Hornet wt. ratio	Flight performance				
ID	Fresh wt. (g)	Type (battery)	Active	Weight (g)		Date	In flight cage		On field release	
							Rating	Category	Max. dist. (m)	Category
W15	0.321	Pip19 (Ag190)	No	0.203	0.63	25/07/17	5	Good	> 50	Good
W16	0.335	Pip19 (Ag190)	Yes	0.224	0.67	26/07/17	(not assessed)		< 2	Poor
W17	0.460	Pip19 (Ag190)	Yes	0.225	0.49	27/07/17	(not assessed)		> 50 *	Good
W32	0.475	PicoPip (Ag337)	Yes	0.305	0.64	13/09/17	(not assessed)		528 *	Good
W39	0.317	Dummy	No	0.338	1.07	15/09/17	1	Poor	30	Good
W34	0.379	Dummy	No	0.360	0.95	15/09/17	2	Poor	(not assessed)	
W37	0.378	Dummy	No	0.352	0.93	15/09/17	3	Poor	23	Good
W35	0.291	Dummy	No	0.291	1.00	15/09/17	2	Poor	(not assessed)	
W38	0.370	Dummy	No	0.216	0.58	15/09/17	5	Good	59	Good
W36	0.333	Dummy	No	0.312	0.94	15/09/17	5	Good	37	Good
W40	0.314	Dummy	No	0.244	0.78	15/09/17	3	Poor	3	Poor
W42	0.409	Dummy	No	0.291	0.71	15/09/17	4	Good	27	Good
W43	0.328	Dummy	No	0.269	0.82	15/09/17	1	Poor	8	Poor
W45	0.426	Dummy	No	0.280	0.66	15/09/17	4	Good	28	Good
W44	0.305	Dummy	No	0.230	0.75	15/09/17	1	Poor	58	Good
W41	0.466	Dummy	No	0.334	0.72	15/09/17	4	Good	51	Good
W53	0.422	Dummy	No	0.465	1.10	18/09/17	3	Poor	17	Good
W54	0.286	Dummy	No	0.256	0.90	18/09/17	5	Good	72	Good
W52	0.291	Dummy	No	0.267	0.92	18/09/17	2	Poor	12	Poor
W48	0.303	Dummy	No	0.262	0.86	18/09/17	2	Poor	(not assessed)	
W46	0.402	Dummy	No	0.473	1.18	18/09/17	2	Poor	4	Poor
W50	0.326	Dummy	No	0.254	0.78	18/09/17	5	Good	(not assessed)	
W47	0.344	Pip19 (Ag190)	No	0.210	0.61	18/09/17	5	Good	(not assessed)	
W49	0.335	Pip19 (Ag190)	No	0.217	0.65	18/09/17	5	Good	16	Good
W51	0.294	Dummy	No	0.273	0.93	18/09/17	4	Good	22	Good
W55	0.258	Dummy	No	0.347	1.34	18/09/17	3	Poor	3	Poor
W57	0.233	Pip19 (Ag190)	No	0.233	1.00	18/09/17	1	Poor	14	Poor

Supplementary Table 1 (continued). Tagged worker hornet's flight performance.

ID	Fresh wt. (g)	Type (battery)	Active	Weight (g)	Tag:Hornet wt. ratio	Date	In flight cage		On field release	
							Rating	Category	Max. dist. (m)	Category
W56	0.233	Pip19 (Ag190)	No	0.219	0.94	18/09/17	2	Poor	13	Good
Y21	0.300	Pip19 (Ag190)	Yes	0.215	0.72	19/09/17	2	Poor	< 5	Poor
W58	0.387	PicoPip (Ag337)	Yes	0.276	0.71	19/09/17	5	Good	(not assessed)	
W59	0.356	PicoPip (Ag337)	Yes	0.281	0.79	19/09/17	5	Good	351 *	Good
W60	0.376	PicoPip (Ag337)	Yes	0.276	0.73	20/09/17	4	Good	1331 *	Good
W61	0.401	PicoPip (Ag337)	Yes	0.278	0.69	20/09/17	4	Good	54 *	Good
W62	0.431	PicoPip (Ag337)	Yes	0.280	0.65	20/09/17	5	Good	195 *	Good
W64	0.412	Dummy	No	0.494	1.20	21/09/17	3	Poor	52	Good
W63	0.328	Dummy	No	0.291	0.89	21/09/17	3	Poor	12	Good
W29	0.229	Dummy	No	0.195	0.85	21/09/17	2	Poor	13	Poor
W65	0.305	Dummy	No	0.269	0.88	21/09/17	3	Poor	5	Poor
W69	0.368	PicoPip (Ag337)	Yes	0.285	0.77	21/09/17	4	Good	343 *	Good
W67	0.341	PicoPip (Ag337)	Yes	0.286	0.84	21/09/17	2	Poor	–	Poor
FG59	0.35	PicoPip (Ag337)	Yes	0.28	0.80	25/09/17	3	Poor	–	Poor
FG1	0.34	PicoPip (Ag337)	Yes	0.28	0.82	25/09/17	3	Poor	–	Poor
G1	0.49	PicoPip (Ag337)	Yes	0.28	0.57	26/09/17	3	Poor	–	Poor
G2	0.47	Pip19 (Ag190)	Yes	0.23	0.49	26/09/17	4	Good	> 32 *	Good
G3	0.36	PicoPip (Ag337)	Yes	0.28	0.77	27/09/17	1	Poor	(not assessed)	
G4	0.41	PicoPip (Ag337)	Yes	0.28	0.68	27/09/17	5	Good	> 40 *	Good
G5	0.46	PicoPip (Ag337)	Yes	0.28	0.61	28/09/17	4	Good	`238 *	Good

Flight performance assessed in the flight cage was rated on a scale of 1-5 (1 = sharp descending flights or no flights; 2 = mainly descending flights; 3 = both horizontal & descending flights; 4 = mainly horizontal flights; 5 = ascending & strong horizontal flights); hornets rated 4 or 5 were categorised as 'Good flyers'. Flight performance on field release was similarly categorised as 'Poor' or 'Good' based on whether flights were largely descending or ascending, respectively. On occasions, when hornets landed in a nearby tree (12-16 m from release point), flight performance was judged on performance prior to that point. Distances indicated equate to the maximum distance from release to point of landing, to last visual sighting of a flying hornet (e.g. perimeter wall), or – if tracked (indicated by *) – to location where tracked to.

Supplementary Table 2. Tracked hornets' summary information.

Hornet ID	Tag type	Date	Caught		Released				Destination				Summary values		
			Latitude	Longitude	Time	Latitude	Longitude	Compass	Time	Latitude	Longitude	Descript.	D (m)	B °	Duration
W17	Pip19	27/07/17	44.787529	-0.576049	14:42	44.787825	-0.576114	125°				Lost			
W32	PicoPip	13/09/17	44.787564	-0.576039	16:45	44.787745	-0.575876	112°	17:30	44.785378	-0.570071	Nest	528	119	0h 45m
W59	PicoPip	19/09/17	44.791409	-0.575086	16:00	44.791409	-0.575086	140°	17:36	44.790567	-0.570806	Nest	351	105	1h 36m
W60	PicoPip	20/09/17	44.789711	-0.580583	11:43	44.789549	-0.580870	310°	19:42	44.789903	-0.597734	Nest	1331	271	2h 13m
W61	PicoPip	20/09/17	44.789711	-0.580583	16:21	44.789469	-0.580578	2°	18:09	44.789742	-0.580093	Conifer	49	50	0h 53m
W62	PicoPip	20/09/17	44.789711	-0.580583	16:12	44.789469	-0.580578	2°	17:15	44.790149	-0.582859	Nest	195	293	1h 03m
W69	PicoPip	21/09/17	44.787564	-0.576039	15:43	44.787762	-0.575937	240°	20:00	44.788455	-0.571700	Garden	343	76	1h 59m
G2	Pip19	26/09/17	49.185790	-2.091615	17:52	49.185690	-2.091641	245°				Lost			
G4	PicoPip	27/09/17	49.225644	-2.074863	16:18	49.224035	-2.074863	350°	09:20	49.224438	-2.070461	Recovered	45	352	1h 11m
G5	PicoPip	28/09/17	49.222725	-2.073565	11:42	49.223419	-2.073400	150°	13:43	49.225433	-2.072273	Nest	238	20	2h 01m

Ten tagged hornets were radio tracked; 7 in Bordeaux (ID starting with W) and 3 in Jersey (ID starting with G). Hornets were caught either hawking outside hives, foraging for nectar or honeydew, or foraging at artificial bait stations. The hornets were fitted with either Pip19 or PicoPip radio-telemetry tags (see Supplementary Table 1 for tag and hornet weights) and released at a nearby open location permitting vanishing compass directions (= Compass) of departing hornets to be recorded. They were tracked by confirming the direction of strongest signal from consecutive waypoints along the approximate route of travel, or either-side of it, until triangulation from various points suggested a stationary location, the hornet or radio-tag were found, or the nest was found. Both hornets fitted with Pip19 tags flew relatively quickly beyond the range of detection and were lost. Of those fitted with PicoPip tags: W61 was tracked to a conifer where signal was detected the rest of the day (presumed caught amongst the fine needles of the tree; no nest observed) but was lost the following day; W69 was tracked to a private residential garden and signal was detected late into the evening and the following morning (as not accessible, unable to check for presence of nest, hornet or tag); G4 was caught in a rain storm shortly after released, sheltered in a beech tree overnight but was found wet & torpid in a nearby field the following morning and recovered; all remaining hornets were successfully tracked to their previously unknown nests. Summary values provided are displacement distance (D) between release point and recorded destination, compass bearing (B) from release point to destination, and length of time (hours & minutes) actually tracking a hornet.

Supplementary Table 3. Tracked hornets' waypoint information.

Each of the ten tagged hornets, referred to in Supplementary Table 2, were radio-tracked from a release point near where they were originally caught. Their flight paths were tracked by moving in the approximate direction of the previous strongest signal, to the extent that terrain and public rights of way permitted this. Signal direction and observational notes were recorded at various waypoints along the tracked route until triangulation from various points suggested a stationary location, the hornet or radio-tag were found, or the hornet's nest was found. In some instances, the location of a tagged hornet near a nest was checked on following days. When tracking of an individual was interrupted (to work on other hornets, for refreshment breaks, etc.) this is indicated in the notes.

(a) Hornet ID = White 17 (W17). Caught hawking outside beehive at the INRA apiary on 27/07/2017. Hornet fresh weight = 0.460 g; Pip19 radio tag = 0.225 g.

Date	Time	Tracked duration	Reference or waypoint (WP)	Latitude	Longitude	Compass direction(s) of detected signal	Notes
27/07/2017	10:00 – 11:00		Capture location	44.787529	-0.576049		
27/07/2017	14:42	00:00	Release location	44.787825	-0.576114	125°	Released at INRA apiary
27/07/2017	14:59		WP 1.2	44.786086	-0.574837	(no signal)	
27/07/2017	15:12		WP 1.3	44.786451	-0.571537	(no signal)	
27/07/2017	15:16		WP 1.4	44.786077	-0.572945	(no signal)	
27/07/2017	15:23		WP 1.5	44.786657	-0.573293	(no signal)	
27/07/2017	15:42		WP 1.6	44.784176	-0.567820	(no signal)	Abandoned as lost

Supplementary Table 3 (continued). Tracked hornets' waypoint information.

(b) Hornet ID = White 32 (W32). Caught hawking outside beehive at the INRA apiary on 13/09/2017. Hornet fresh weight = 0.475 g; PicoPip radio tag = 0.305 g.

Date	Time	Tracked duration	Reference or waypoint (WP)	Latitude	Longitude	Compass direction(s) of detected signal	Notes
13/09/2017	11:00 – 12:00		Capture location	44.787564	-0.576039		
13/09/2017	16:45	00:00	Release location	44.787745	-0.575876	112°	Released at INRA apiary
13/09/2017	–	–	Opposite playground	44.786816	-0.574659	28° & 63°	Hornet on the move
13/09/2017	17:07	00:22	WP 2.3	44.787165	-0.572595	14° & 173°	Reflections off buildings?
13/09/2017	17:11	00:26	WP 2.4	44.786975	-0.571873	30°	Signal across properties
13/09/2017	17:15	00:30	WP 2.5	44.788239	-0.572002	138°	Signal more southerly
13/09/2017	17:17	00:32	WP 2.6	44.787676	-0.571789	166°	Hornet moving?
13/09/2017	17:20	00:35	WP 2.7	44.787021	-0.571286	152°	Signal more southerly
13/09/2017	17:25	00:40	WP 2.8	44.786607	-0.570797	168°	Signal more southerly
13/09/2017	17:27	00:42	WP 2.9	44.786422	-0.570557	158°	Signal more southerly
13/09/2017	17:28	00:43	WP 2.10	44.785856	-0.570454	136°	Signal to south-east; follow footpath towards tower block
13/09/2017	17:30	00:45	WP 2.11	44.785474	-0.570094	182°	Spot nest high in tree approx. 10m in front of WP 2.11
13/09/2017			Nest location	44.785378	-0.5700706		Estimated GPS location based on visually judged distance from WP 2.11
13/09/2017	17:32		WP 2.12	44.785424	-0.569491	262°	Confirmed signal from nest
14/09/2017	09:15		WP 2.11	44.785474	-0.570094	182°	Signal still from nest
14/09/2017	14:10		WP 2.11	44.785474	-0.570094	(no signal)	Signal not at nest; W32 left
14/09/2017	14:35		Sweet chestnut tree; tag found	44.787663	-0.577256		Signal detected; tag found under tree frequented by foraging hornets
15/09/2017	11:00		Apiary	44.787745	-0.575876		Recaught W32 hornet hawking outside beehive at INRA apiary

Supplementary Table 3 (continued). Tracked hornets' waypoint information.

(c) Hornet ID = White 59 (W59). Caught at bait station within INRA grounds on 19/09/2017. Hornet fresh weight = 0.356 g; PicoPip radio tag = 0.281 g.

Date	Time	Tracked duration	Reference or waypoint (WP)	Latitude	Longitude	Compass direction(s) of detected signal	Notes
19/09/2017	13:35		Capture location	44.791409	-0.575086		
19/09/2017	16:00	00:00	Release location	44.791409	-0.575086	140°	Towards INRA's eastern boundary.
19/09/2017	16:14	00:14	WP 3.2	44.788606	-0.572833	356°	Signal more northerly
19/09/2017	16:19	00:19	WP 3.3	44.789603	-0.572534	322°	Signal more northerly
19/09/2017	16:27	00:27	WP 3.4	44.791204	-0.573380	153°	Overshot; signal along boundary wall to south
19/09/2017	16:38	00:38	WP 3.5	44.790355	-0.573235	50°	Close but W59 then moves
19/09/2017	16:39	00:39	Flowering ivy	44.790049	-0.573160	–	Suspect W59 foraging here
19/09/2017	16:40	00:40	WP 3.6	44.790061	-0.573180	58°	Near ivy; then moves
19/09/2017	16:54	00:54	WP 3.7	44.789539	-0.572327	298°	Back towards ivy?
19/09/2017	16:57	00:57	WP 3.8	44.789698	-0.572433	58°	Previous reflection off building
19/09/2017	17:02	01:02	WP 3.9	44.790521	-0.571210	52°	Signal from private garden
19/09/2017	17:04	01:04	WP 3.10	44.790946	-0.571332	130°	Triangulating
19/09/2017	17:06	01:06	WP 3.11	44.790997	-0.570949	180°	Triangulating
19/09/2017	17:36	01:36	WP 3.12	44.790534	-0.571226	80°	Nest spotted; hornet traffic
19/09/2017			Nest location	44.790567	-0.570806		Triangulated location

20/09/2017	09:05		WP 3.12	44.790534	-0.571226		Signal still from nest
20/09/2017	14:43		WP 3.13	44.790469	-0.571209	76°	Signal still from nest
20/09/2017	14:46		WP 3.14	44.790830	-0.571296	138°	Signal still from nest
20/09/2017	14:49		WP 3.15	44.790295	-0.571177	30°	Signal still from nest
20/09/2017	18:30		WP 3.16	44.787924	-0.576798	46°	Signal still from nest
21/09/2017	09:05		WP 3.12				Signal still from nest
22/09/2017	07:57		WP 3.12				Signal still from nest but weak

Supplementary Table 3 (continued). Tracked hornets' waypoint information.

(d) Hornet ID = White 60 (W60). Caught foraging at willow tree within INRA grounds on 20/09/2017. Hornet fresh weight = 0.376 g; PicoPip radio tag = 0.276 g.

Date	Time	Tracked duration	Reference or waypoint (WP)	Latitude	Longitude	Compass direction(s) of detected signal	Notes
20/09/2017	10:00		Caught location	44.789711	-0.580583		
20/09/2017	11:43	00:00	Release location	44.789549	-0.580870	310°	Flew into shrubs & trees
20/09/2017	11:50	00:07	WP 4.2	44.789519	-0.581163	334°	
20/09/2017	12:03	00:20	WP 4.3	44.790251	-0.582204	121°	Overshot; amongst trees
20/09/2017	12:04	00:21	WP 4.4	44.79005	-0.581773	114°	
20/09/2017	12:05	00:22	WP 4.5	44.789985	-0.581555	106°	
20/09/2017	12:07	00:24	WP 4.6	44.789939	-0.581443	126°	
20/09/2017	12:13	00:30	WP 4.7	44.789603	-0.581267	93°	Amongst a cluster of trees
20/09/2017	12:15	00:32					Break for lunch
20/09/2017	13:46	00:32					Renew search
20/09/2017	13:48	00:34	WP 4.8	44.789601	-0.580920	138°	
20/09/2017	13:52	00:38	WP 4.9	44.789449	-0.580761	196°	W60 moving
20/09/2017	13:54	00:40	WP 4.10	44.789190	-0.580860		
20/09/2017	13:56	00:42	WP 4.11	44.789080	-0.581038		
20/09/2017	14:12	00:58	Mulberry tree				Foraging on tree within WP 4.10, WP 4.11 & WP 4.12
20/09/2017	14:13	00:59	WP 4.12	44.789060	-0.580875	300°	
20/09/2017	14:15	01:01					Break; work on other hornets
20/09/2017	16:15	01:01					Return to tracking W60
20/09/2017	16:19	01:05	WP 4.13			260°	
20/09/2017	16:20	01:06					Break while tracking W61
20/09/2017	16:25	01:06					Return to tracking W60
20/09/2017	16:25	01:06	WP 4.14	44.78967	-0.580677	240°	Signal still nearby
20/09/2017	16:30	01:11					Break; tracking W61 & W62

Supplementary Table 3 (continued). Tracked hornets' waypoint information.

(d) (W60 continued).

Date	Time	Tracked duration	Reference or waypoint (WP)	Latitude	Longitude	Compass direction(s) of detected signal	Notes
20/09/2017	18:30						Return to tracking W60
20/09/2017	18:30	01:11	WP 4.14	44.789670	-0.580677	(no signal)	Finish for the day?
20/09/2017	18:40	01:11	La Cave de Pascal	44.785811	-0.581727	300°	Detect faint signal
20/09/2017	19:00	01:31	WP 4.16	44.787626	-0.585642	266°	Signal more westerly
20/09/2017	19:03	01:34	WP 4.17	44.787880	-0.586491	276°	Signal more westerly
20/09/2017	19:12	01:43	WP 4.18	44.788510	-0.589333	288°	Signal more westerly
20/09/2017	19:19	01:50	WP 4.19	44.789138	-0.592787	282°	In park 'Bois de Thouars'
20/09/2017	19:21	01:52	WP 4.20	44.789337	-0.593530	280°	
20/09/2017	19:26	01:57	WP 4.21	44.789416	-0.595122	304° & 310°	
20/09/2017	19:29	02:00	WP 4.22	44.789880	-0.596342	240°	
20/09/2017	19:35	02:06	WP 4.23	44.789736	-0.596641	320°	
20/09/2017	19:37	02:08	WP 4.24	44.789657	-0.597568	0°	
20/09/2017	19:38	02:09	WP 4.25	44.789782	-0.597730	30°	
20/09/2017	19:39	02:10	WP 4.26	44.789818	-0.597834	73°	
20/09/2017	19:42	02:13	Nest location	44.789903	-0.597734		Nest in oak tree directly above
21/09/2017	09:05		Nest location	44.789903	-0.597734		Signal still at nest

Supplementary Table 3 (continued). Tracked hornets' waypoint information.

(e) Hornet ID = White 61 (W61). Caught foraging at willow tree within INRA grounds on 20/09/2017. Hornet fresh weight = 0.401 g; PicoPip radio tag = 0.278 g.

Date	Time	Tracked duration	Reference or waypoint (WP)	Latitude	Longitude	Compass direction(s) of detected signal	Notes
20/09/2017	14:15		Capture location	44.789711	-0.580578		
20/09/2017	16:21	00:00	Release location	44.789469	-0.580578	2°	Flew back to capture location
20/09/2017	16:25	00:04	WP 5.2	44.789670	-0.580677	204°	Seen W61 depart from willow tree and fly in 208° direction
20/09/2017	16:30	00:09	WP 5.3	44.789157	-0.581202	86°	
20/09/2017	16:32	00:11	WP 5.4	44.789131	-0.580740	281°	
20/09/2017	16:35	00:14					Break while tracking W62
20/09/2017	17:30	00:14					Return to tracking W61
20/09/2017	17:30	00:14	WP 5.5	44.789506	-0.580840	274°	
20/09/2017	18:09	00:53	WP 5.6	44.789756	-0.580091	214°	W61 tracked to cedar tree; no visual; tag snagged?
20/09/2017	18:10		Atlas cedar tree	44.789742	-0.580093		Tree location
21/09/2017	10:06		WP 5.7	44.789742	-0.580091	(no signal)	
21/09/2017	10:13		WP 5.8	44.791636	-0.575231	(no signal)	
21/09/2017	10:19		WP 5.9	44.787902	-0.576644	(no signal)	Abandoned as lost

Supplementary Table 3 (continued). Tracked hornets' waypoint information.

(f) Hornet ID = White 62 (W62). Caught foraging at willow tree within INRA grounds on 20/09/2017. Hornet fresh weight = 0.431 g; PicoPip radio tag = 0.280 g.

Date	Time	Tracked duration	Reference or waypoint (WP)	Latitude	Longitude	Compass direction(s) of detected signal	Notes
20/09/2017	14:15		Capture location	44.789711	-0.580583		
20/09/2017	16:12	00:00	Release location	44.789469	-0.580578	2°	Flew back to capture location
20/09/2017	16:15	00:03	Willow tree	44.789695	-0.580566		
20/09/2017	16:25	00:13	WP 6.3	44.789670	-0.580677	70°	
20/09/2017	16:36	00:24	WP 6.4	44.789440	-0.580723	308°	Flew over nearby building
20/09/2017	16:40	00:28	WP 6.5	44.790477	-0.581124	252°	
20/09/2017	16:41	00:29	WP 6.6	44.790581	-0.581519	290°	
20/09/2017	16:43	00:31	WP 6.7	44.790896	-0.581659	224°	Signal more easterly
20/09/2017	16:52	00:40	WP 6.8	44.790414	-0.582330	240°	Signal from in Parc Sourreil
20/09/2017	16:56	00:44	WP 6.9	44.790208	-0.582486	290°	
20/09/2017	16:56	00:44	WP 6.10	44.790338	-0.583148	140°	
20/09/2017			Nest location	44.790149	-0.582859		Nest high in oak tree in Parc Sourreil

21/09/2017	09:45		Nest location	44.790149	-0.582859		Signal no longer at nest
21/09/2017	09:51		WP 6.12	44.790070	-0.582946	183°	Signal detected but changing
21/09/2017	09:53		WP 6.13	44.789540	-0.583118	167°	
21/09/2017	09:53		WP 6.13	44.789540	-0.583118	62°	
21/09/2017	09:58		WP 6.14	44.789441	-0.581920	332°	W62 evidently moving

Supplementary Table 3 (continued). Tracked hornets' waypoint information.

(g) Hornet ID = White 69 (W69). Caught hawking outside beehive at the INRA apiary on 21/09/2017. Hornet fresh weight = 0.368 g; PicoPip radio tag = 0.285 g.

Date	Time	Tracked duration	Reference or waypoint (WP)	Latitude	Longitude	Compass direction(s) of detected signal	Notes
21/09/2017	14:00		Capture location	44.787564	-0.576039		
21/09/2017	15:43	00:00	Release location	44.787762	-0.575937	240°	Flew to chestnut tree
21/09/2017	15:49	00:06	Sweet chestnut tree	44.787677	-0.576053		
21/09/2017	16:10	00:27	WP 7.3	44.787750	-0.576179	293°	Switched trees
21/09/2017	16:11	00:28	WP 7.4	44.787788	-0.576400	126°	
21/09/2017	16:13	00:30					Break while return to lab
21/09/2017	16:56	00:30					Return to tracking W69
21/09/2017	16:58	00:32	WP 7.5	44.787857	-0.576221	120°	Signal to south-east
21/09/2017	17:00	00:34	WP 7.6	44.787533	-0.575501	128°	
21/09/2017	17:07	00:41	WP 7.7	44.787075	-0.574857	336°	Signal from tree
21/09/2017	17:09	00:43	WP 7.8	44.787457	-0.574979	203°	W69 seen flying around tree
21/09/2017	17:10	00:44	WP 7.9	44.787312	-0.575102	190°	Foraging?
21/09/2017	17:11	00:45	WP 7.10	44.787196	-0.575215	52°	Foraging?
21/09/2017	17:13	00:47	WP 7.11	44.787256	-0.575008		W69 seen flying around tree
21/09/2017	17:15	00:49					Break while return to lab
21/09/2017	18:50	00:49					Return to tracking W69
21/09/2017	18:50	00:49	WP 7.12	44.785892	-0.570021	223°	Not at W32's nest location
21/09/2017	18:51	00:50	WP 7.13	44.785790	-0.570436	2°	Signal more north
21/09/2017	18:53	00:52	WP 7.14	44.786426	-0.570537	326°	Signal more north
21/09/2017	18:58	00:57	WP 7.15	44.786839	-0.571537	278°	
21/09/2017	19:07	01:06	WP 7.16	44.787676	-0.571738	30°	
21/09/2017	19:09	01:08	WP 7.17	44.788036	-0.571357	229°	
21/09/2017	19:12	01:11	WP 7.18	44.788236	-0.571883	132°	Signal from private garden
21/09/2017	20:00	01:59	Private garden	44.788455	-0.571700		No access to check for nest

Supplementary Table 3 (continued). Tracked hornets' waypoint information.

(g) (W69 continued).

Date	Time	Tracked duration	Reference or waypoint (WP)	Latitude	Longitude	Compass direction(s) of detected signal	Notes
22/09/2017	07:33		WP 7.20	44.788106	-0.571331	220°	Signal still from same private garden
22/09/2017	07:37		WP 7.21	44.787733	-0.571213	292°	Signal still from same private garden
22/09/2017	07:45		WP 7.22	44.788914	-0.572859	136°	Signal still from same private garden; unable to gain access

Supplementary Table 3 (continued). Tracked hornets' waypoint information.

(h) Hornet ID = Green 2 (G2). Caught in hornet trap near St Savoir, Jersey on 26/09/2017. Hornet fresh weight = 0.47 g; Pip19 radio tag = 0.23 g.

Date	Time	Tracked duration	Reference or waypoint (WP)	Latitude	Longitude	Compass direction(s) of detected signal	Notes
26/09/2017	16:20		Capture location	49.185790	-2.091615		
26/09/2017	17:52	00:00	Release location	49.185690	-2.091641	245°	Initial orientation flight; then flew in 245° direction
26/09/2017	17:54	00:02	WP 8.2	49.185688	-2.091821		Seen circling tree
26/09/2017	18:05	00:13	WP 8.3	49.185673	-2.091934	260°	Left tree and flew over wall
26/09/2017	18:06		WP 8.4	49.185654	-2.092096	(no signal)	
26/09/2017	18:09		WP 8.5	49.185730	-2.093681	(no signal)	Abandoned as lost

Supplementary Table 3 (continued). Tracked hornets' waypoint information.

(i) Hornet ID = Green 4 (G4). Caught at bait station south of Durrell Zoo, Jersey, on 27/09/2017. Hornet fresh weight = 0.41 g; Pip19 radio tag = 0.28 g.

Date	Time	Tracked duration	Reference or waypoint (WP)	Latitude	Longitude	Compass direction(s) of detected signal	Notes
27/09/2017	15:10		Capture location	49.225644	-2.074863		
27/09/2017	16:18	00:00	Release location	49.224035	-2.070408	350°	Released at wrong (not near capture) location in error
27/09/2017	16:21	00:03	WP 9.2	49.224446	-2.070860	50°	
27/09/2017	16:23	00:05	WP 9.3	49.224521	-2.070597	37°	Starts to rain; amongst trees
27/09/2017	16:29	00:11	WP 9.4	49.224832	-2.069843	216° & 228°	Light rain continues
27/09/2017	16:33	00:15	WP 9.5	49.224610	-2.070851	90	Within trees at field edge
27/09/2017	16:38	00:20	WP 9.6	49.224611	-2.071013	290	Conflicting signals; reflection?
27/09/2017	16:51	00:33	WP 9.7	49.222967	-2.066871	307° & 172°	Relocate for clearer signal
27/09/2017	16:56	00:38	WP 9.8	49.222690	-2.068691	(no clear signal)	Heavy rain
27/09/2017	17:17	00:59	Beech tree	49.224673	-2.070461		Strong signal at beech tree
27/09/2017	20:30		Beech tree	49.224673	-2.070461		Heavy rain; still at beech tree; break for the night
28/09/2017	07:38	00:59	Beech tree	49.224673	-2.070461		Renew tracking of G4
28/09/2017	07:45	01:06	WP 9.10	49.224438	-2.070498		Signal from patch in meadow
28/09/2017	07:45	01:06					Break to collect colleague
28/09/2017	09:15	01:06					Renew tracking of G4
28/09/2017	09:20	01:11	WP 9.10	49.224438	-2.070498		G4 found & collected from patch in meadow
							G4 dried & fed
28/09/2017	11:26	01:11	WP 9.11	49.225767	-2.075194		Released at correct (near capture) location; G4 refuses to fly; attempt abandoned

Supplementary Table 3 (continued). Tracked hornets' waypoint information.

(j) Hornet ID = Green 5 (G5). Caught at another bait station south of Durrell Zoo, Jersey, on 27/09/2017. Hornet fresh weight = 0.46 g; Pip19 radio tag = 0.28 g.

Date	Time	Tracked duration	Reference or waypoint (WP)	Latitude	Longitude	Compass direction(s) of detected signal	Notes
27/09/2017	16:30.		Capture location	49.222725	-2.073565		G5 held overnight in small cage with *ad libitum* food
28/09/2017	11:42	00:00	Release location	49.223419	-2.073400	150°	
28/09/2017	12:07	00:25	WP 10.2	49.223203	-2.073210		Landed in grass/clover field
28/09/2017	12:09	00:27	WP 10.3	49.223207	-2.073204	146°	Assisted & then flew off
28/09/2017	12:23	00:41	WP 10.4	49.222670	-2.073128	190°	South of capture location
28/09/2017	12:26	00:44	WP 10.5	49.222346	-2.072862	346°	
28/09/2017	12:31	00:49	WP 10.6	49.222329	-2.072873	56° & 203°	Confusing signals; swap Yagi antenna & Sika receiver
28/09/2017	12:44	01:02	WP 10.7	49.222314	-2.072894	356°	Clear signal
28/09/2017	12:45	01:03	WP 10.8	49.222608	-2.072771	308°	In trees; then moving away
28/09/2017	13:00	01:18	WP 10.9	49.224003	-2.072936	3°	Flowering ivy covered tree
28/09/2017	13:02	01:20	WP 10.10	49.224161	-2.072950	183°	Foraging on ivy?
28/09/2017	13:05	01:23	WP 10.11	49.224155	-2.072948	42°	G5 seen flying away from tree
28/09/2017	13:10	01:28	WP 10.12	49.224415	-2.072482	7°	
28/09/2017	13:13	01:31	WP 10.13	49.224609	-2.072407	346°	
28/09/2017	13:25	01:43	WP 10.14	49.225832	-2.072097	209° - 241°	Signal more south-west
28/09/2017	13:29	01:47	WP 10.15	49.225619	-2.073572	72°	Signal more east
28/09/2017	13:32	01:50	WP 10.16	49.225566	-2.072885	73° - 127°	Signal more east
28/09/2017	13:34	01:52	WP 10.17	49.225517	-2.072633	120°	Signal from private garden
28/09/2017	13:42	02:00	WP 10.18	49.225406	-2.072229	134° - 166°	Given permission to enter garden by owner
28/09/2017	13:43	02:01	WP 10.19	49.225410	-2.072080	232°	Nest spotted
28/09/2017			Nest location	49.225433	-2.072273		Nest in silver birch in garden
